# Is single-inhaler triple therapy for COPD cost-effective in the UK? The IMPACT trial

**DOI:** 10.1183/23120541.00333-2021

**Published:** 2022-02-21

**Authors:** Alan Martin, Dhvani Shah, Kerigo Ndirangu, Glenn A. Anley, Gabriel Okorogheye, Melanie Schroeder, Nancy Risebrough, Afisi S. Ismaila

**Affiliations:** 1 Value Evidence and Outcomes, GlaxoSmithKline, Uxbridge, UK; 2 ICON Plc, Health Economics, New York, NY, USA; 3 Value Evidence and Outcomes, GlaxoSmithKline, Brentford, UK; 4 ICON Plc, Health Economics, Toronto, ON, Canada; 5 Value Evidence and Outcomes, GlaxoSmithKline, Collegeville, PA, USA; 6 Dept of Health Research Methods, Evidence and Impact, McMaster University, Hamilton, ON, Canada

## Abstract

**Background:**

The IMPACT trial demonstrated superior outcomes following 52 weeks of once-daily single-inhaler treatment with fluticasone furoate (FF)/umeclidinium (UMEC)/vilanterol (VI) (100/62.5/25 μg) compared with once-daily FF/VI (100/25 μg) or UMEC/VI (62.5/25 μg). This study evaluated the cost-effectiveness of FF/UMEC/VI compared with FF/VI or UMEC/VI for the treatment of chronic obstructive pulmonary disease (COPD) from a UK National Health Service perspective.

**Methods:**

Patient characteristics and treatment effects from IMPACT were populated into a hybrid decision tree/Markov economic model. Costs (GB£ inflated to 2018 equivalents) and health outcomes were modelled over a lifetime horizon, with a discount rate of 3.5% per annum applied to both. Sensitivity analyses were performed to test the robustness of key assumptions and input parameters.

**Results:**

Compared with FF/VI and UMEC/VI, FF/UMEC/VI provided an additional 0.296 and 0.145 life years (LYs) (discounted) and 0.275 and 0.118 quality-adjusted life years (QALYs), at an additional cost of £1129 and £760, respectively. Incremental cost-effectiveness ratios (ICERs) for FF/UMEC/VI were £4104/QALY and £3809/LY gained *versus* FF/VI and £6418/QALY and £5225/LY gained *versus* UMEC/VI. At a willingness-to-pay threshold of £20 000/QALY, the probability that FF/UMEC/VI was cost-effective was 96% *versus* FF/VI and 74% *versus* UMEC/VI. Results were similar in a subgroup of patients recommended triple therapy in the 2019 National Institute for Health and Care Excellence COPD guideline.

**Conclusions:**

FF/UMEC/VI single-inhaler triple therapy improved health outcomes and was a cost-effective option compared with FF/VI or UMEC/VI for patients with symptomatic COPD and a history of exacerbations in the UK at recognised cost-effectiveness threshold levels.

## Abstract 2

**This analysis demonstrates that fluticasone furoate (FF)/umeclidinium (UMEC)/vilanterol (VI) provides a cost-effective treatment option *versus* FF/VI or UMEC/VI for patients with symptomatic COPD in the UK** https://bit.ly/3w7vQj9

## Introduction

The Global Initiative for Chronic Obstructive Lung Disease (GOLD) has noted that the healthcare costs associated with chronic obstructive pulmonary disease (COPD) are substantial and increase with disease severity [1]. Patients who have advanced disease represent a subgroup of the COPD patient population typically associated with greater healthcare resource utilisation (HRU) [1].

GOLD recommends triple pharmacological therapy, comprising an inhaled corticosteroid (ICS), a long-acting β_2_-agonist (LABA) and a long-acting muscarinic antagonist (LAMA), for patients with COPD who remain symptomatic or at risk of exacerbations despite treatment with dual regimens [1]. Similarly, the National Institute for Health and Care Excellence (NICE) 2019 guideline on COPD recommended the use of triple therapy for patients receiving ICS/LABA if their day-to-day symptoms continue to adversely impact their quality of life, and for patients on either ICS/LABA or LAMA/LABA who experience a severe exacerbation requiring hospitalisation or two moderate exacerbations within a year [2]. Historically, triple therapy has been prescribed through multiple inhaler combination therapies; however, single-inhaler triple therapies (SITT) are now available [3–8]. There is currently a paucity of economic analyses comparing the use of SITT with dual therapies in the UK [9–11].

The phase III InforMing the PAthway of COPD Treatment (IMPACT) trial was a randomised, double-blind, parallel-group, multicentre study [3, 12]. IMPACT demonstrated superior exacerbation reduction and lung function improvement over 52 weeks of once-daily single-inhaler ICS/LAMA/LABA treatment with a combination of fluticasone furoate (FF) (100 μg), umeclidinium (UMEC) (62.5 μg) and vilanterol (VI) (25 μg), compared with FF/VI (100/25 μg) or UMEC/VI (62.5/25 μg). The rate of COPD-related hospitalisations was also lower amongst those treated with FF/UMEC/VI compared with those who received UMEC/VI [3].

The aim of this study was to evaluate the cost-effectiveness of FF/UMEC/VI SITT compared with dual therapy with either FF/VI or UMEC/VI for the treatment of COPD, using data from the IMPACT clinical trial, from a UK National Health Service (NHS) perspective.

## Methods

### Cost-effectiveness model structure

**FIGURE 1:**
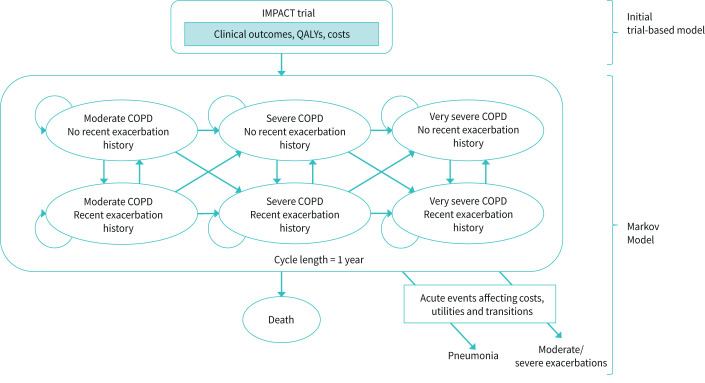
Conceptual COPD disease progression model. Reproduced from [11] with permission. Moderate COPD (FEV_1_ % pred 50–<80%); severe COPD (FEV_1_ % pred 30–<50%); very severe COPD (FEV_1_ % pred <30%). A recent exacerbation history is defined as an exacerbation occurring within the previous cycle. QALY: quality-adjusted life year; COPD: chronic obstructive pulmonary disease; FEV_1_: forced expiratory volume in 1 s.

The economic model used has been published previously [11]. Briefly, it comprised two parts: an initial decision tree representing clinical outcomes directly from IMPACT results for the 1-year trial period, and a Markov model to extrapolate outcomes over the longer term (figure 1). The six health states in the Markov model are stratified according to recent exacerbation history (exacerbation/no exacerbation within the previous 12 months) and three categories of COPD severity. The categories of COPD severity were defined by per cent predicted forced expiratory volume in 1 s (FEV_1_ % pred) based on GOLD classifications (moderate: 50–<80%, severe: 30–<50% and very severe: <30%) [11].

Patients started the Markov model in health states according to the distribution observed at the end of the IMPACT trial. The movement of patients to more severe states (FEV_1_ decline) and exacerbation risk in each state (exacerbation risk increases with COPD severity and with a history of exacerbation within the previous 12 months) was predicted by risk equations, estimated using data from the 3-year Towards a Revolution in COPD Health (TORCH) study [13], because no data beyond 52 weeks were available from the IMPACT trial. Annual transition probabilities and annual risk of exacerbation by severity state are shown in supplementary table S1. Health-related quality of life (HRQoL) and HRU were assigned to health states (with more severe states having higher HRU and poorer HRQoL), and disutilities were applied to exacerbation and pneumonia events.

### Model inputs

#### Patient population

Patient characteristics from the IMPACT trial intent-to-treat (ITT) population were used for this analysis [3]. A subgroup reflecting patients recommended for triple therapy in the 2019 NICE COPD guideline was also examined [2]. Patients eligible for inclusion in the IMPACT study were aged ≥40 years with symptomatic advanced COPD, current or former smokers with a smoking history of ≥10 pack years, and had at least one moderate or severe exacerbation during the previous 12 months [3, 12].

Mean patient age was 65.3 years, with patients being predominantly male (66%). The distribution of baseline characteristics was similar across all three treatment groups, including the incidence of previous COPD exacerbations. Baseline patient characteristics from the TORCH trial (supplementary table S2) [13] were used to generate the risk equations for the annual FEV_1_ decline and exacerbation rates.

### Comparators

The comparators included in the model analysis were FF/UMEC/VI (100/62.5/25 μg), FF/VI (100/25 μg) and UMEC/VI (62.5/25 μg). These treatments were all administered once daily using the single-dose ELLIPTA inhaler (GlaxoSmithKline, Middlesex, UK).

### Treatment effect

Treatment effect at 52 weeks in the IMPACT ITT population, by treatment arm, was used. For lung function, this was included as the change in distribution across COPD severity (defined by FEV_1_ % pred) health states during the trial period, with trial end defining patient distribution at the beginning of the Markov phase. No further effect on lung function was included, and FEV_1_ declined in all patients at the rate for the health state occupied in each cycle.

Treatment effect on exacerbation reduction was incorporated directly during the within-trial period, and then, in the base case, indirectly in the longer term through more patients distributed to less severe COPD Markov states, or states without a recent exacerbation, which have a lower risk of exacerbation. In scenario analysis, directly including the exacerbation rate reduction with FF/UMEC/VI observed in IMPACT in the longer term was explored. Exacerbation rates from the IMPACT study [3] and health distributions at trial end/Markov initiation are shown in supplementary table S3.

### Treatment discontinuation

Treatment discontinuation was included in the initial decision tree part of the model, using data obtained from the IMPACT trial at end of follow-up. Subsequent treatment discontinuation in the Markov model period was not included due to a lack of long-term data. It was therefore assumed that patients remain on comparator treatment for COPD for the duration of time in the Markov model. Because patients were allowed to remain in the IMPACT trial after discontinuing therapy, the impact on treatment effect was considered to be accounted for in the ITT results, and discontinuation was assumed to only affect costs. Treatment costs were calculated assuming that patients who discontinued did so at the midpoint of the trial (*i.e.* they received 26 weeks of trial-assigned treatment and subsequently 26 weeks of replacement therapy). It was assumed that patients who discontinued remained on replacement therapy for the duration of the analysis time horizon. The cost of replacement therapy was based on the proportion of patients who received any of the four most common COPD medication classes used after discontinuation in IMPACT. In the base case, treatment arm-specific replacement treatment distributions were used; in the scenario analyses, the pooled replacement therapy distributions across all three treatment arms were applied across all initial treatments.

### Pneumonia

Pneumonia was included as the only adverse event considered to potentially impact outcomes of the analysis. The rate of pneumonia was as reported for the IMPACT ITT population and in additional *post hoc* analyses for the subgroup; in IMPACT, pneumonia incidence was analysed as a safety end-point, and thus separately from the annual rate of moderate or severe exacerbations (primary end-point in IMPACT). The rate of pneumonia was assumed to be dependent only on the treatment received, irrespective of COPD severity (supplementary table S3). Pneumonia mortality was assumed to be accounted for by the overall COPD excess mortality rates.

### Mortality

Mortality occurring during the trial period was included within the base case and was as reported in the IMPACT trial (supplementary table S3). In the Markov model, mortality was estimated using COPD severity-specific risks (table 1), relative to patients without COPD, derived from a study of COPD mortality rates [20]. These were applied to rates from UK general population life tables [22] adjusted to exclude reported COPD deaths in the UK [23].

**TABLE 1:** Markov model health state utilities [14], costs and QALY loss associated with each event [15]

**Parameter**	**Input**	**Source**
**Health state costs (total per annum)**		[15–19]
Moderate COPD	£216.82	
Severe COPD	£798.95	
Very severe COPD	£2297.98	
**Relative risk of mortality**		[20]
Moderate COPD	1.89	
Severe COPD	3.63	
Very severe COPD	8.33	
**Exacerbation costs (total per event)**		[14–19]
Severe exacerbation	£6120.30	
Moderate exacerbation	£568.48	
Pneumonia^#^	£1087.98	
**Drug cost per 30 days**		[17]
FF/UMEC/VI	£44.50	
FF/VI	£22.00	
UMEC/VI	£32.50	
**Health state utilities, utility (95% CI)**		[14]
Moderate COPD	0.787 (0.771–0.802)	
Severe COPD	0.750 (0.731–0.786)	
Very severe COPD	0.647 (0.598–0.695)	
**Event disutilities, QALY loss per event (95% CI)**		[21]
Severe exacerbation	0.020 (0.020–0.030)	
Moderate exacerbation	0.011 (0.006–0.020)	
Pneumonia^¶^	0.011 (0.006–0.020)	

COPD was classified as moderate (FEV_1_ % pred 50–<80%), severe (FEV_1_ % pred 30–<50%) or very severe (FEV_1_ % pred <30%). QALY: quality-adjusted life year; COPD: chronic obstructive pulmonary disease; FF: fluticasone furoate; UMEC: umeclidinium; VI: vilanterol; CI: confidence interval; FEV_1_: forced expiratory volume in 1 s. ^#^: weighted cost calculated using ambulatory and inpatient costs; ^¶^: assumption, equivalent to moderate exacerbation.

### Costs

Health state, exacerbation and drug costs are provided in table 1. All costs are in GB£ 2018 (inflated to 2018 costings using the Consumer Price Index [24] where applicable (supplementary table S4)). Drug costs were sourced from the Monthly Index of Medical Specialities [17].

HRU estimates for maintenance care at each level of COPD severity, and management of exacerbations, were taken from the available literature [18] and HRU unit cost from NHS reference costs (inflated to 2018) [15] and Personal Social Services Research Unit costs (inflated to 2018) [16] (supplementary table S4). For the cost of managing/treating pneumonia, ambulatory and inpatient unit costs from NHS references [15, 16] were weighted by the proportion of pneumonia cases hospitalised during the IMPACT trial (55%) [3], giving a weighted cost of £1087.98 per pneumonia event.

Costs of replacement therapies were based on weighted average use in 2018 UK market share data. Costs were estimated for the most commonly used therapies post-discontinuation in IMPACT within each of the four therapy classes: £29.29 for LAMA monotherapy, £31.79 for ICS/LABA, £32.50 for LAMA/LABA and £61.08 for ICS/LAMA/LABA (personal communication). The distribution of replacement therapies by class in IMPACT was similar for each treatment arm, resulting in 30-day replacement costs of £48.01, £48.54 and £49.34 for FF/UMEC/VI, UMEC/VI and FF/VI, respectively.

Societal costs (*e.g.* productivity losses due to absenteeism) were estimated according to the human capital approach, which can be broadly interpreted as estimating the lost gross value during time absent from usual activities [25], whether or not this means formal or paid employment. Societal costs were only included in the scenario analyses.

### Health-related quality of life

During the trial period, utilities were calculated directly from EQ-5D data collected in IMPACT using the UK value set. The pooled baseline EQ-5D score was used for all treatments; treatment-specific utilities were then calculated by adding change from baseline in EQ-5D score at each time point where data were collected. Utilities for health states and disutility for exacerbations and pneumonia for the Markov model were sourced from the literature (table 1) [2, 14].

### Modelling assumptions

Only moderate and severe exacerbations were included, because it was assumed that mild exacerbations do not have a significant impact on clinical and economic outcomes. In the Markov model, it was assumed that individuals can only transition to increasingly severe health states, given that COPD is a progressive disease.

### Analyses

#### Base case

The base case analysis was conducted in the IMPACT ITT population over a lifetime (35 years) time horizon with costs and outcomes discounted at 3.5% per annum as per NICE guidance [26].

#### Subgroup analyses

In addition to the IMPACT ITT population, a subgroup analysis was conducted in the population of patients identified in the NICE COPD 2019 guidelines [2] as being appropriate for triple therapy, *i.e.* patients who experienced at least two moderate exacerbations or at least one severe exacerbation requiring hospitalisation in the previous year. IMPACT results for the subgroup are shown in supplementary table S3.

#### Scenario analyses

Scenario analyses were conducted with alternative model settings for discount rates, within-trial mortality, treatment discontinuation, time horizon, replacement therapy distribution, inclusion of societal costs and within-trial utility. Because the base case conservatively assumed no FF/UMEC/VI treatment effect on exacerbations beyond the trial period, scenario analyses were also conducted assuming treatment effect up to 5 years, both with constant full effect and with waning to zero, over that period.

#### Sensitivity analyses

Deterministic one-way sensitivity analyses were performed, varying a range of input parameters by ±20%, including the utility associated with very severe or moderate COPD, exacerbation rates, risk of mortality, costs of FF/UMEC/VI, FF/VI and UMEC/VI, and COPD maintenance costs (supplementary table S5).

A probabilistic sensitivity analysis (PSA) was also conducted, with random sampling from distributions assigned to input parameters over 10 000 Monte Carlo simulations. Distributions used in the PSA are shown in supplementary table S6. Risk equation coefficients were included in the PSA *via* Cholesky decomposition. The findings were summarised as incremental cost-effectiveness ratio (ICER) scatter plots and cost-effectiveness acceptability curves.

## Results

**FIGURE 2:**
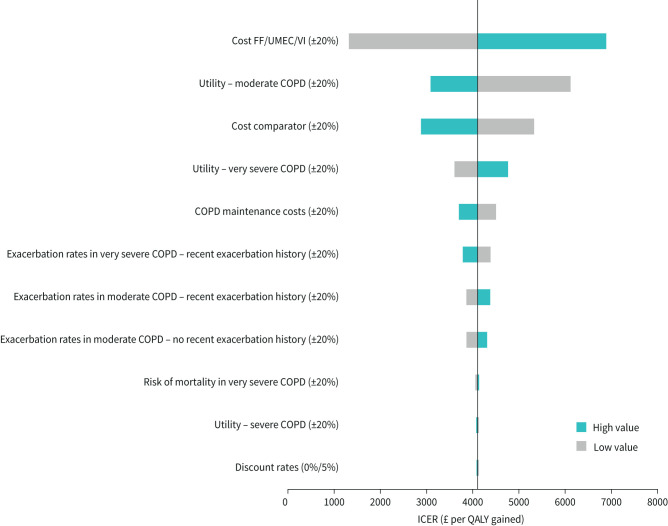
One-way sensitivity analysis plot for FF/UMEC/VI compared with FF/VI (QAYLs; ITT population). Moderate COPD (FEV_1_ % pred 50–<80%); severe COPD (FEV_1_ % pred 30–<50%); very severe COPD (FEV_1_ % pred <30%). ICER: incremental cost-effectiveness ratio.

**FIGURE 3:**
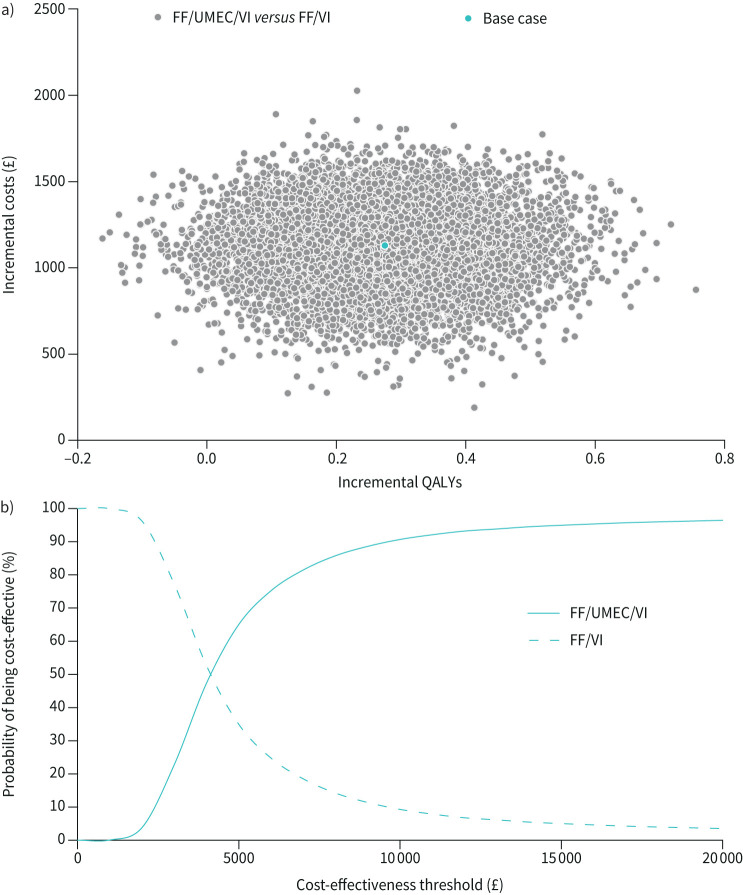
a) ICER scatter plot (QALYs) and b) cost-effectiveness acceptability curve for FF/UMEC/VI compared with FF/VI. ICER: incremental cost-effectiveness ratio; QALY: quality-adjusted life year; FF: fluticasone furoate; UMEC: umeclidinium; VI: vilanterol.

Compared with FF/VI, FF/UMEC/VI provided an additional 0.296 (95% CI 0.198–0.399) life years (LYs) (discounted) and 0.275 (0.033–0.512) quality-adjusted life years (QALYs), at an additional cost of £1129 (£683–£1533) (table 2), over the 35-year time horizon. The ICER in the base case was £4104 (£1646–£19 201) per QALY gained and £3809 (£2199–£6177) per LY gained (table 2). In one-way sensitivity analyses, ICERs ranged from £1320 to £6888 (figure 2), with results most sensitive to the cost of FF/UMEC/VI, utility associated with moderate COPD and the cost of FF/VI. In scenario analyses, ICERs ranged from dominant (when the time horizon was restricted to the trial follow-up period) to £6234 (with no treatment discontinuation within or post trial) (table 3). In the PSA *versus* FF/VI, FF/UMEC/VI improved health outcomes and was more costly in the majority of simulations (figure 3a), with a probability of being cost-effective of 96%, at a willingness-to-pay threshold of £20 000 per QALY (figure 3b) [27].

**TABLE 2:** Results for FF/UMEC/VI compared with FF/VI or UMEC/VI base case ITT population and NICE recommended subgroup

	**FF/UMEC/VI**	**FF/VI**	**Incremental (FF/UMEC/VI *versus* FF/VI)**	**UMEC/VI**	**Incremental (FF/UMEC/VI *versus* UMEC/VI)**
**Base case ITT population**					
**Predicted exacerbations**					
Moderate exacerbations	5.712	5.799	–0.087	5.888	–0.176
Severe exacerbations	1.371	1.378	–0.007	1.420	–0.049
Any moderate and/or severe exacerbation	7.083	7.177	–0.094	7.309	–0.225
**Total LYs (discounted)**	8.874	8.577	0.296 (0.198–0.399)	8.728	0.145 (0.041–0.253)
**Total QALYs**	6.564	6.289	0.275 (0.033–0.512)	6.446	0.118 (–0.124–0.355)
**Costs**					
Maintenance	£7926	£8479	–£552	£8030	–£104
Moderate exacerbations	£2666	£2738	–£72	£2776	–£110
Severe exacerbations	£6827	£6929	–£102	£7154	–£327
Pneumonia	£963	£940	£22	£606	£357
Treatment	£3881	£1759	£2122	£2546	£1335
Replacement therapy	£806	£1095	–£290	£1197	–£392
**Total costs**	£23 069	£21 941	£1129 (£683–£1533)	£22 310	£760 (£305–£1165)
**ICER per LY gained**			£3809 (£2199–£6177)		£5225 (£1704–£19 702)
**ICER per QALY gained**			£4104 (£1646–£19 201)		£6418 (dominant, £65 705)
**Patients with ≥2 moderate or ≥1 severe exacerbation in the previous year**					
**Predicted exacerbations**					
Moderate exacerbations	5.784	5.862	–0.078	5.983	–0.199
Severe exacerbations	1.384	1.396	–0.012	1.464	–0.080
Any moderate and/or severe exacerbation	7.168	7.258	–0.090	7.447	–0.279
**Total LYs (discounted)**	9.142	8.895	0.247 (0.148–0.356)	8.996	0.146 (0.029–0.266)
**Total QALYs**	6.800	6.571	0.229 (−0.013–0.473)	6.686	0.114 (−0.134–0.365)
**Costs**					
Maintenance	£7468	£7942	–£474	£7562	–£94
Moderate exacerbations	£2688	£2750	–£62	£2811	–£123
Severe exacerbations	£6856	£6978	–£122	£7369	–£514
Pneumonia	£1012	£965	£47	£640	£371
Treatment	£4026	£1843	£2183	£2624	£1402
Replacement therapy	£806	£1084	–£278	£1222	–£417
**Total costs**	£22 855	£21 562	£1293 (£873–£1686)	£22 228	£627 (£122–£1076)
**ICER per LY gained**			£5235 (£2997–£9662)		£4289 (£524–£21 488)
**ICER per QALY gained**			£5642 (dominant, £37 302)		£5495 (dominant, £61 459)

Outcomes and costs observed over the complete period (trial-based and Markov models). All ranges are 95% confidence intervals, unless otherwise stated. 95% confidence intervals were derived from the probabilistic sensitivity analysis. FF: fluticasone furoate; UMEC: umeclidinium; VI: vilanterol; ITT: intention-to-treat; NICE: National Institute for Health and Care Excellence; LY: life year; QALY: quality-adjusted life year; ICER: incremental cost-effectiveness ratio.

**TABLE 3:** Scenario analyses (FF/UMEC/VI *versus* FF/VI or UMEC/VI) in the ITT population

	**Base case**	**Scenario**	**FF/UMEC/VI *versus* FF/VI (ICER/QALY gained)**	**FF/UMEC/VI *versus* UMEC/VI (ICER/QALY gained)**
**Base case**			£4104	£6418
**Discount rates (costs, benefits)**	3.5%	0.0%	£4134	£6731
**Discount rates (costs, benefits)**	3.5%	5.0%	£4082	£6227
**Within-trial mortality**	Included	Excluded	£4132	£8123
**Post-trial treatment effect:**^#^ **no waning**	No direct effect	Direct effect for 1 year	£3293	£3301
**Post-trial treatment effect: no waning**	No direct effect	Direct effect for 3 years	£1951	Dominant
**Post-trial treatment effect: no waning**	No direct effect	Direct effect for 5 years	£891	Dominant
**Post-trial treatment effect: with waning**	No direct effect	Direct effect for 5 years	£1993	Dominant
**Treatment discontinuation**	Within-trial treatment discontinuation applied	No treatment discontinuation within or post trial	£6234	£9455
**Time horizon**	Lifetime	Trial follow-up	Dominant	Dominant
**Replacement therapy**	Replacement therapy specific per initial treatment	Average replacement therapy across all initial treatments	£4104	£6418
**Perspective**	Health service perspective	Societal perspective^¶^	£3442	£3739
**Utility for within-trial period**	EQ-5D data from IMPACT	Health state-specific utility data [14]	£4051	£6301

FF: fluticasone furoate; UMEC: umeclidinium; VI: vilanterol; ITT: intent-to-treat; ICER: incremental cost-effectiveness ratio; QALY: quality-adjusted life year. ^#^: the post-trial treatment effect analyses applied the relative risk reductions in exacerbations observed during the IMPACT trial; ^¶^: indirect costs include productivity losses incurred by absenteeism, estimated according to the human capital approach, which can be broadly interpreted as estimating the lost gross value during time absent from usual activities.

**FIGURE 4:**
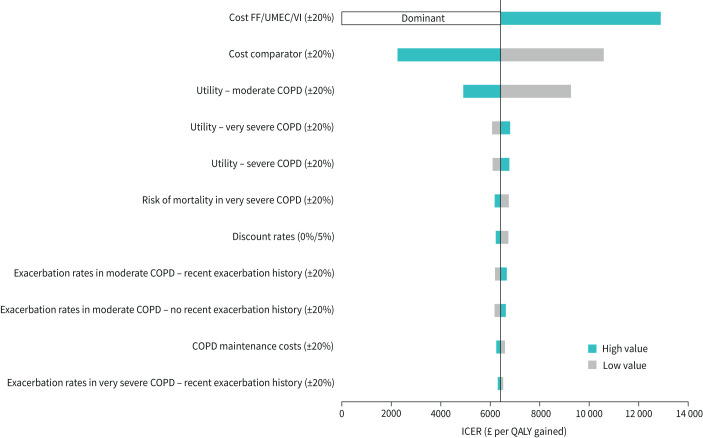
One-way sensitivity analysis plot for FF/UMEC/VI compared with UMEC/VI (QALYs; ITT population). Moderate COPD (FEV_1_ % pred 50–<80%); severe COPD (FEV_1_ % pred 30–<50%); very severe COPD (FEV_1_ % pred <30%). FF: fluticasone furoate; UMEC: umeclidinium; VI: vilanterol; QALY: quality-adjusted life year; ITT: intent-to-treat; COPD: chronic obstructive pulmonary disease; FEV_1_: forced expiratory volume in 1 s; ICER: incremental cost-effectiveness ratio.

**FIGURE 5:**
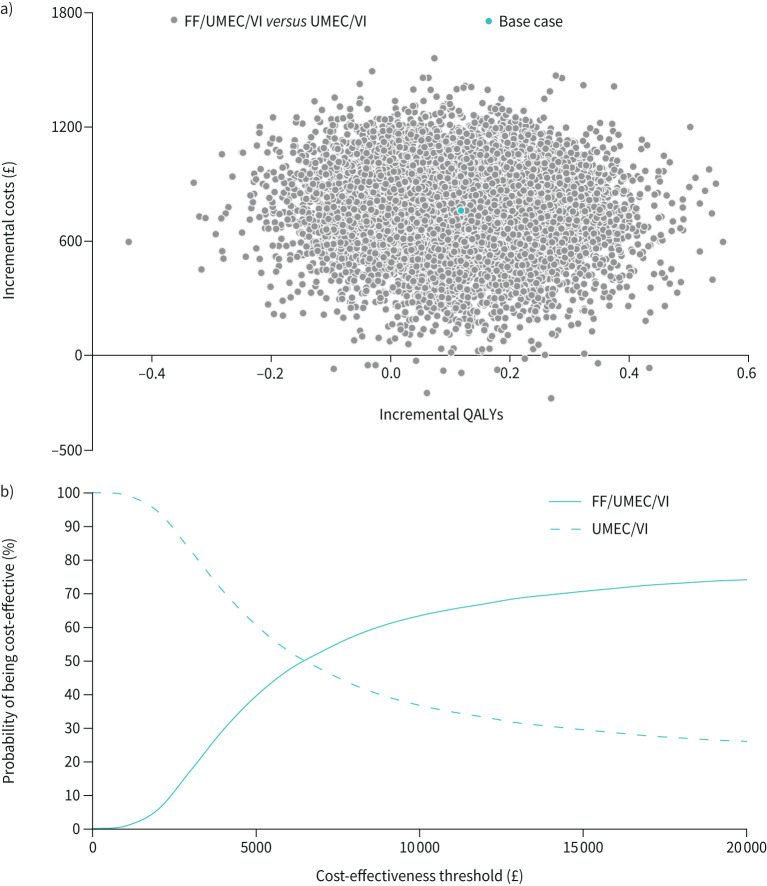
a) ICER scatter plot (QALYs) and b) cost-effectiveness acceptability curve for FF/UMEC/VI compared with UMEC/VI. ICER: incremental cost-effectiveness ratio; QALY: quality-adjusted life year; FF: fluticasone furoate; UMEC: umeclidinium; VI: vilanterol.

Compared with UMEC/VI, FF/UMEC/VI provided an additional 0.145 (0.041–0.253) LYs (discounted) and 0.118 (–0.124–0.355) QALYs, at an additional cost of £760 (£305–£1165) (table 2). This resulted in an ICER of £6418 (dominant (greater benefits at lower cost), £65 705) per QALY gained and £5225 (£1704–£19 702) per LY gained (table 2). In one-way sensitivity analyses, these results were shown to be most sensitive to the cost of both FF/UMEC/VI and UMEC/VI, and the utility associated with moderate COPD (figure 4); ICERs ranged from dominant to £12 888. Also, FF/UMEC/VI remained cost-effective when compared with UMEC/VI across all scenarios (table 3). In PSA, FF/UMEC/VI was associated with improved health outcomes and was more costly than UMEC/VI in the majority of simulations (figure 5a) with a probability of being cost-effective compared with UMEC/VI of 74% at a willingness-to-pay threshold of £20 000 per QALY gained (figure 5b).

In the subgroup of patients who would be eligible to receive triple therapy according to NICE guideline recommendations [2], results were similar compared with the base case (table 2). The ICERs were £5642 (dominant, £37 302) per QALY gained and £5235 (£2997–£9662) per LY gained compared with FF/VI and £5495 (dominant, £61 459) per QALY gained and £4289 (£524–£21 488) per LY gained compared with UMEC/VI.

## Discussion

This analysis of IMPACT data from the UK NHS perspective provides strong evidence to support the cost-effectiveness of prescribing FF/UMEC/VI for the treatment of patients with COPD who remain symptomatic or at risk of exacerbations, despite treatment with dual regimens, in the UK. FF/UMEC/VI remained cost-effective across all sensitivity and scenario analyses and, importantly, FF/UMEC/VI was shown to be cost-effective compared with FF/VI or UMEC/VI in those patients for whom NICE guidelines [2] recommend the use of inhaled triple therapy.

These findings are consistent with evidence from other economic evaluations of FF/UMEC/VI. A study using the Galaxy model [28] to compare FF/UMEC/VI with budesonide/formoterol (BUD/FOR) dual therapy (based on data from the FULFIL trial) also demonstrated that FF/UMEC/VI is a cost-effective option for the treatment of patients with symptomatic COPD from a UK NHS perspective [29]. This analysis was repeated using the current model, as part of a comparison of the two models presented at ISPOR 2019 [30], with similar estimates of cost-effectiveness from both models. Similar findings from a Spanish National Healthcare System perspective have also been reported; a Galaxy model analysis using data from the FULFIL trial demonstrated that FF/UMEC/VI was cost-effective compared with BUD/FOR dual therapy over a 3-year time horizon [10]. In a study in a Canadian setting, also using the Galaxy model and based on data from the IMPACT study, FF/UMEC/VI was shown to be cost-effective compared with FF/VI and UMEC/VI over a lifetime horizon from a Canadian healthcare system perspective [9].

The Galaxy model uses linked risk equations to predict long-term outcomes, the equations taking account of some baseline patient clinical characteristics in addition to treatment effect on FEV_1_, exacerbations and St George's Respiratory Questionnaire scores. The model used in this analysis, although including two risk equations, uses a Markov approach, predicting outcomes based on FEV_1_, exacerbation history and age alone. Applying different modelling approaches for the same decision problem strengthens the evidence base and provides important corroborating evidence where results indicate a similar conclusion of cost-effectiveness. The current model design was chosen because the Markov approach is familiar to many reimbursement and health technology agencies. In addition, the economic analysis conducted in support of the most recent NICE COPD guideline employed a Markov model [2]. A table comparing the features of the models can be found in supplementary table S7.

Total costs were higher with FF/UMEC/VI than either comparator, largely as a result of the higher acquisition cost of FF/UMEC/VI. This was offset to some extent by savings in medical management costs with FF/UMEC/VI, resulting from fewer exacerbations and less time spent in the more severe COPD states where HRU is higher. The potential for a reduction in healthcare costs with FF/UMEC/VI is corroborated by an analysis of HRU in the FULFIL study [31] and a US-based within-trial economic analysis of HRU costs in the IMPACT trial [32]. Of the two comparisons analysed in this paper**,** the ICER was lower for FF/UMEC/VI than for UMEC/VI, despite total costs being lower with FF/VI than UMEC/VI. This is because outcomes in the Markov model are driven primarily by the distribution across health states defined by FEV_1_ % pred. This results in the FF/VI arm (single bronchodilator) having the lowest QALYs, because FF/VI shows the least improvement in FEV_1_, and consequently QALY gains are higher for FF/UMEC/VI *versus* FF/VI, compared with FF/UMEC/VI *versus* UMEC/VI (0.275 *versus* 0.118, respectively).

It should be noted that any potential effect of baseline eosinophil levels on cost-effectiveness of treatment was not examined in this analysis. In the prespecified analysis of the annual rate of moderate or severe exacerbations conducted within IMPACT, the observed reduction in exacerbations with triple therapy *versus* dual therapy was statistically significant regardless of baseline eosinophil level [3]. Moreover, at the present time, discussions are ongoing regarding the most appropriate cut-off points for eosinophil count analyses [33]. Once these have been determined, it may then be appropriate for this to be considered within cost-effectiveness models.

Limitations of this study include the fact that, common to all analyses of cost-effectiveness using clinical trial data, results may not be generalisable to clinical practice. In addition, no long-term data (beyond 52 weeks) were available from the IMPACT study and thus, for long-term outcomes, risk equations based on data from the TORCH trial (with a 3-year follow-up) were used. While the populations in the two trials were broadly similar, whether the results of TORCH are fully generalisable to the IMPACT population is not certain. However, the model-projected reduction in predicted exacerbations with FF/UMEC/VI post trial, based on lung function, was observed to be substantially smaller than that seen in IMPACT, and is therefore unlikely to over-estimate the benefit of FF/UMEC/VI. Scenario analyses showed that the results were sensitive to assumptions on post-trial exacerbation treatment effect, with a substantial reduction in the ICER if the trial effects were applied to longer time horizons. Restricting treatment discontinuation to the trial period may also be a limitation. It is reasonable to assume that the majority of discontinuation occurs early in treatment, but longer-term data on discontinuation patterns would be desirable. However, in all sensitivity and scenario analyses, FF/UMEC/VI was cost-effective, suggesting that uncertainty around long-term effects does not substantially affect the study conclusion. Finally, comorbidity is not explicitly modelled in this analysis, despite the fact that it is likely to influence mortality risk as well as the severity and consequent healthcare costs of exacerbations and pneumonia in an individual patient. However, this is unlikely to affect analysis results for the following reason: IMPACT was a randomised study with a very large sample size, therefore distribution of comorbidities, and the consequent impact on mortality and healthcare costs, should be similar across treatments. Hence, comorbidity should not impact treatment comparisons of cost, health outcomes and cost-effectiveness.

In addition to the robustness of results to sensitivity analysis, a strength of this study is that comparative efficacy and safety data were derived from a study (IMPACT) in which all treatments evaluated consisted of the same component molecules administered *via* the same inhaler, and at the same dosing frequency. This uniquely allows for strict evaluation of the benefit of triple therapy with FF/UMEC/VI compared with dual therapy with LAMA/LABA or ICS/LABA. However, this does mean that any potential benefits of SITT compared with multiple-inhaler triple therapy (*e.g.* ease of use or adherence), or of once-daily dosing over more frequent dosing regimens, were not reflected in the analysis, and must be the subject of further research.

In conclusion, FF/UMEC/VI SITT was predicted to improve health outcomes and be a cost-effective option when compared with FF/VI or UMEC/VI for patients with symptomatic COPD and a history of exacerbations in the UK, at recognised cost-effectiveness threshold levels and in line with NICE COPD guidelines.

## Supplementary material

10.1183/23120541.00333-2021.Supp1 **Please note:** supplementary material is not edited by the Editorial Office, and is uploaded as it has been supplied by the author.
Supplementary material 00333-2021.SUPPLEMENT
