# Supplementary material for: Is single-inhaler triple therapy for COPD cost-effective in the UK? The IMPACT trial
Source: ERJ Open Res. 2022 Feb 21;8(1):00333-2021. doi: 10.1183/23120541.00333-2021 (PMC8859506; doi:10.1183/23120541.00333-2021)
Supplement: Supplementary file 1 [file 00333-2021.SUPPLEMENT.pdf]

Supplementary appendix

SUPPLEMENTARY TABLE S1 Annual transition probabilities (based on statistical equations for FEV_1 decline over time)

COPD severity health state transition	Transition probability
Moderate COPD to severe COPD (no recent exacerbation history)	4.11%
Moderate COPD to severe COPD (recent exacerbation history)	8.83%
Severe COPD to very severe COPD (no recent exacerbation history)	7.83%
Severe COPD to very severe COPD (recent exacerbation history)	14.28%

Moderate COPD (FEV_1 percent predicted 50–<80%); severe COPD (FEV_1 percent predicted 30–<50%); very severe COPD (FEV_1 percent predicted <30%). A recent exacerbation history is defined as an exacerbation occurring within the previous cycle.

FEV_1: forced expiratory volume in 1 second; COPD: chronic obstructive pulmonary disease.

SUPPLEMENTARY TABLE S2 Baseline patient characteristics

Parameter		Source
**Sex**		
Male	75.8%	TORCH [1]
**Age category, years**		
<55	11.5%	TORCH, data on file
55–<65	32.3%	TORCH, data on file
65–<75	43.7%	TORCH, data on file
≥75	12.6%	TORCH, data on file
**Exacerbation history (moderate or severe, in the previous 12 months)**		
0	43.0%	TORCH, data on file
1	24.8%	TORCH, data on file
≥2	32.3%	TORCH, data on file
**BMI, kg/m^2**		
<20	13.5%	TORCH, data on file
20–<25	37.6%	TORCH, data on file
25–<29	26.9%	TORCH, data on file
≥29	22.0%	TORCH, data on file
**SGRQ total score**		
<38	28.8%	TORCH, data on file
38–<50	25.7%	TORCH, data on file
50–<62	22.9%	TORCH, data on file
≥62	22.6%	TORCH, data on file

BMI: body mass index; SGRQ: St. George’s Respiratory Questionnaire.

SUPPLEMENTARY TABLE S3 Model inputs: trial outcomes and health-state distribution at end of trial/start of Markov model

	**FF/UMEC/VI**	**FF/VI**	**UMEC/VI**
**<u>IMPACT ITT population</u>**			
**Trial outcomes (on-treatment event analysis)**			
Annual rate of moderate COPD exacerbations (95% CI)	0.75 (0.71–0.79)	0.89 (0.85–0.93)	0.97 (0.91–1.04)
Annual rate of severe COPD exacerbations (95% CI)	0.13 (0.12–0.14)	0.15 (0.13–0.16)	0.19 (0.17–0.22)
Pneumonia (rate/1000 patient-years)	95.8	96.6	61.2
**Deaths (n, %)[#]**	70 (1.7)	78 (1.9)	50 (2.4)
**Utility inputs**			
Baseline (pooled)[¶]	0.788	0.788	0.788
28-week change from baseline	0.017	0.014	0.013
52-week change from baseline	0.013	0.006	0.002
**Health-state distribution at end of trial/start of Markov model**			
**No within-trial exacerbations (%)**			
Moderate COPD (FEV_1 percent predicted 50–<80%)	27.7	23.1	25.2
Severe COPD (FEV_1 percent predicted 30–<50%)	19.6	19.5	21.1
Very severe COPD (FEV_1 percent predicted <30%)	5.2	7.5	6.0
**With within-trial exacerbations (%)**			
Moderate COPD (FEV_1 percent predicted 50–<80%)	17.3	13.9	16.6
Severe COPD (FEV_1 percent predicted 30–<50%)	19.9	23.0	20.5
Very severe COPD (FEV_1 percent predicted <30%)	8.6	11.1	8.1
**Treatment discontinuation (%)**	18.3	25.2	27.3
**<u>IMPACT subgroup: ≥2 moderate exacerbations or ≥1 severe exacerbation</u>**			
**Trial outcomes**			
Annual rate of within-trial moderate COPD exacerbations	0.77 (0.73–0.81)	0.88 (0.84–0.94)	1.01 (0.94–1.10)

	FF/UMEC/VI	FF/VI	UMEC/VI
Annual rate of within-trial severe COPD exacerbations	0.13 (0.11–0.14)	0.15 (0.13–0.17)	0.22 (0.19–0.25)
Pneumonia (rate/1000 patient-years)[+]	97.9	95.8	62.9
**Deaths (n, %)[#]**	31 (1.1)	34 (1.2)	27 (1.9)
**Utility inputs**			
Baseline (pooled)	0.788	0.788	0.788
28-week change from baseline	0.018	0.016	0.017
52-week change from baseline	0.015	0.011	0.010
**Health-state distribution at end of trial/start of Markov model**			
**No within-trial exacerbations (%)**			
Moderate COPD (FEV_1 percent predicted 50–<80%)	31.8%	28.1%	29.7%
Severe COPD (FEV_1 percent predicted 30–<50%)	15.6%	16.6%	15.8%
Very severe COPD (FEV_1 percent predicted <30%)	4.0%	5.1%	4.2%
**With within-trial exacerbations (%)**			
Moderate COPD (FEV_1 percent predicted 50–<80%)	21.4%	17.5%	20.3%
Severe COPD (FEV_1 percent predicted 30–<50%)	18.0%	21.7%	20.3%
Very severe COPD (FEV_1 percent predicted <30%)	8.1%	9.8%	7.8%
**Treatment discontinuation (%)**	17.6	24.0	27.3

FF: fluticasone furoate; UMEC: umeclidinium; VI: vilanterol; ITT: intent-to-treat; COPD: chronic obstructive pulmonary disease; CI: confidence interval; FEV_1: forced expiratory volume in 1 second.

[#]: From adjudicated fatal serious adverse events; [¶]: Pooled utility for all three treatment arms in the IMPACT trial was used at baseline; [+]: GlaxoSmithKline, data on file.

SUPPLEMENTARY TABLE S4 Itemised resource use and unit costs for COPD management and exacerbations

Cost category	Resource use (per annum)[#]	Unit cost (2018)[+]	Overall cost
**Moderate COPD management**			
Outpatient visit GP [2]	2.00	£39.01	£78.02
Spirometry [3]	2.00	£66.40	£132.81
Influenza vaccination [4]	0.75	£8.00	£6.00
**Total cost of moderate COPD (per annum)**			**£216.82**
**Severe COPD management**			
Outpatient visit, respiratory physician [3]	2.00	£212.39	£424.78
Spirometry [3]	2.00	£66.40	£132.81
Influenza vaccination [4]	0.75	£8.00	£6.00
Oxygen therapy (days) [5]	14.60	£16.12	£235.36
**Total cost of severe COPD (per annum)**			**£798.95**
**Very severe COPD management**			
Outpatient visit RP [3]	4.00	£212.39	£849.56
Spirometry [3]	4.00	£66.40	£265.61
Influenza vaccination [4]	0.75	£8.00	£6.00
Oxygen therapy (days) [5]	73.00	£16.12	£1176.81
**Total cost of very severe COPD (per annum)**			**£2297.98**
	Resource use (per exacerbation)[#]	Unit cost (2018)	Cost (per exacerbation)
**Moderate exacerbation**			
Non-ICU days [3]	1.01	£413.90	£418.04
ER visits [3]	0.03	£221.68	£6.65
Outpatient visit, RP [3]	0.34	£212.39	£72.21
Outpatient visit, GP [2]	0.66	£39.01	£25.75
Visit other healthcare provider [3]	0.27	£153.70	£41.50
Antibiotics[¶] [4]	7.94	£0.45	£3.54
Systemic steroids[¶] [4]	7.94	£0.10	£0.80
**Total cost per moderate exacerbation**			**£568.48**
**Severe exacerbation**			
ICU days [3]	0.86	£1377.43	£1184.59
Non-ICU days [3]	11.08	£413.90	£4586.01
ER visits [3]	0.25	£221.68	£55.42
Outpatient visit, respiratory physician [3]	0.82	£212.39	£174.16
Outpatient visit, GP [2]	0.70	£39.01	£27.31
Visit other healthcare provider [3]	0.50	£153.70	£76.85
Antibiotics[¶] [4]	11.75	£0.9	£10.16
Systemic steroids[¶] [4]	24.08	£0.1	£2.43

Oxygen therapy [5]	0.21	£16.1	£3.39
**Total cost per severe exacerbation**			**£6120.30**

Moderate COPD (FEV_1 percent predicted 50–<80%); severe COPD (FEV_1 percent predicted 30–<50%); very severe COPD (FEV_1 percent predicted <30%).

COPD: chronic obstructive pulmonary disease; GP: general practitioner; ICU: intensive care unit; ER: emergency room; RP: respiratory physician; FEV_1: forced expiratory volume in 1 second.

[#]: Resource use estimates come from OOSTENBRINK et al., 2005 [5]; [¶]: Unit cost represents the cost per days or visit or per category; [+]: All costs were updated to 2018 using the Office on National Statistics inflation and price indices [6].

SUPPLEMENTARY TABLE S5 one-way sensitivity analysis: pre-specified upper and lower limits for pre-selected parameters, Markov model

**Parameter**	**Range**	**Base case**	**Lower limit**	**Upper limit**
Utility associated with very severe COPD	±20%	0.647	0.518	0.776
Utility associated with severe COPD	±20%	0.750	0.600	0.900
Utility associated with moderate COPD	±20%	0.787	0.630	0.944
Exacerbation rates in very severe COPD - recent exacerbation history	±20%	1.200	0.960	1.440
Exacerbation rates in moderate COPD - no recent exacerbation history	±20%	0.299	0.239	0.359
Exacerbation rates in moderate COPD - recent exacerbation history	±20%	0.735	0.588	0.882
Risk of mortality in very severe COPD	±20%	8.33	6.664	9.996
Discount rates for costs and benefits	–	3.5%	0%	5%
Cost of comparator	±20%			
FF/VI		£22.00	£17.60	£26.40
UMEC/VI		£32.50	£26.00	£39.00
Cost FF/UMEC/VI	±20%	£44.50	£35.60	£53.40
COPD maintenance cost	±20%			
Moderate COPD (FEV_1 percent predicted 50–<80%)		£216.82	£173.46	£260.19
Severe COPD (FEV_1 percent predicted 30–<50%)		£798.95	£639.16	£958.74
Very severe COPD (FEV_1 percent predicted <30%)		£2297.98	£1838.38	£2757.57

Moderate COPD (FEV_1 percent predicted 50–<80%); severe COPD (FEV_1 percent predicted 30–<50%); very severe COPD (FEV_1 percent predicted <30%).

COPD: chronic obstructive pulmonary disease; FF: fluticasone furoate; VI: vilanterol; UMEC: umeclidinium; FEV_1: forced expiratory volume in 1 second.

SUPPLEMENTARY TABLE S6 Distributions used in the PSA

**Parameter**	**Distribution**	**Justification**
Patient characteristics[#]	Normal	Assumed normally distributed in the population
COPD mortality rates	Log normal	
Relative risk[¶]	Log normal	Ratio, additive on log scale
Trial-based model probabilities	Beta/Dirichlet	Constrained on interval of 0 to 1
Risk equation coefficients[+]	Multivariate normal with Cholesky decomposition	To capture correlation between normally distributed regression coefficients
Unit costs	Gamma	Constrained on interval of 0 to positive infinity
Resource use rates	Gamma	Constrained on interval of 0 to positive infinity
Resource use probabilities	Beta	Constrained on interval of 0 to 1
Utilities	Beta	Constrained on interval 0 and 1
QALY loss	Gamma	Constrained on interval of 0 to positive infinity

PSA: probabilistic sensitivity analysis; COPD: chronic obstructive pulmonary disease; QALY: quality-adjusted life years; FEV1: forced expiratory volume in 1 second.

[#]: Age, height; [¶]: COPD mortality, exacerbations; [+]: FEV_1 decline, exacerbations.

SUPPLEMENTARY TABLE S7 Comparison of the Markov and GALAXY models

	**Markov**	**GALAXY**
**Structure**	Decision tree followed by Markov model	Linked-risk equations
**Treatment effects**	FEV_1 effect applied at the start of the Markov phase; exacerbation risk determined by FEV_1 status. Direct exacerbation treatment effect not applied	Applied at each annual model cycle to FEV_1, SGRQ score, and moderate/severe exacerbations
**Risk equations**	Based on TORCH [7] and applied to cohort by health state (according to COPD severity)	Based on ECLIPSE [8,9] (clinical) and TORCH [7] (resource use) and applied across the cohort
**Utility**	Derived from EQ-5D data collected in UPLIFT [10] and applied to health states, exacerbation events, and pneumonia events	SGRQ score predicted per annual cycle (based on lung function, recent exacerbations, symptoms, and baseline factors) and then mapped to utility using a validated algorithm [11]
**Disease progression**	Risk equations predict rate of FEV_1 decline (FEV_1 health state transitions) and risk of exacerbations, based on baseline covariates, COPD severity, and history of exacerbations in previous cycle	FEV_1, exacerbations, symptoms, and exercise capacity are predicted by risk equations based on baseline covariates and disease status in previous cycle
**Mortality**	Risk of death derived from mortality tables, with specific health-state excess risk multipliers	Estimated survival in each year based on predicted clinical status that year
**Costs**	Directly assigns HRU (rates from literature [5]) and associated costs to health states and exacerbation and pneumonia events	Costs applied to cohort mean HRU rates predicted by risk equations based on baseline covariates, disease status, and exacerbation events
**Baseline inputs**	**Markov**	**GALAXY**
**Common to both**	Age, sex, BMI, FEV_1, exacerbation history, SGRQ score	
**Differ between models**	BMI, SGRQ, and age categorized differently than in GALAXY to predict FEV_1 decline and exacerbation count, and applied across health states. TORCH population demographics [7] (sex, BMI, SGRQ score, and	FULFIL demographics [12] (CVD comorbidity, other comorbidities, mMRC dyspnea score, current smoking status), estimated 6MWT distance, estimated fibrinogen levels used to predict FEV_1 decline, exacerbation count, SGRQ decline and survival for patient cohort

	baseline exacerbation history) used as a data source	

FEV_1: forced expiratory volume in 1 second; SGRQ: St. George's Respiratory Questionnaire; COPD: chronic obstructive pulmonary disease; EQ-5D, EuroQol-5D health questionnaire; HRU, healthcare resource utilization; BMI, body mass index; CVD, cardiovascular disease; mMRC, modified Medical Research Council; 6MWT, 6-minute walk test.

References

1. Calverley PMA, Anderson JA, Celli B, *et al*. Salmeterol and fluticasone propionate and survival in chronic obstructive pulmonary disease. *New Engl J Med* 2007; 356: 775–789.
2. Personal Social Services Research Unit. Unit Costs of Health and Social Care. www.pssru.ac.uk/project-pages/unit-costs/ Date last accessed: October 16, 2019.
3. National Health Service. National Health Service Reference Costs 2016/17, 2017. https://improvement.nhs.uk/resources/reference-costs/ Date last accessed: October 16, 2019.
4. Monthly Index of Medical Specialities (MIMS), 2019. www.mims.co.uk/ Date last accessed: November 18, 2019.
5. Oostenbrink JB, Rutten-van Mölken MPMH, Monz BU, *et al*. Probabilistic Markov model to assess the costeffectiveness of bronchodilator therapy in COPD patients in different countries. *Value Health* 2005; 8: 32–46.
6. Office for National Statistics. Office for National Statistics inflation and price indices. www.ons.gov.uk/economy/inflationandpriceindices Date last accessed: May 6, 2021.
7. Crim C, Calverley PM, Anderson JA, *et al*. Pneumonia risk in COPD patients receiving inhaled corticosteroids alone or in combination: TORCH study results. Eur Repir J 2009; 34: 641–647.
8. Briggs AH, Baker T, Risebrough NA, *et al*. Development of the GALAXY chronic obstructive pulmonary disease (COPD) model using data from ECLIPSE: internal validation of a linked-equations cohort model. *Med Decis Making* 2017; 37: 469–480.
9. Exuzides A, Colby C, Briggs AH, *et al*. Statistical modelling of disease progression for chronic obstructive pulmonary disease using data from the ECLIPSE study. *Med Decis Making* 2017; 37: 453–468.
10. Tashkin DP, Celli B, Senn S, *et al*. A 4-year trial of tiotropium in chronic obstructive pulmonary disease. *N Engl J Med* 2008; 359: 1543–1554.
11. Starkie HJ, Briggs AH, Chambers MG, *et al*. Predicting EQ-5D values using the SGRQ. *Value Health* 2011; 14: 354–360.
12. Lipson DA, Barnacle H, Birk R, *et al*. FULFIL trial: once-daily triple therapy for patients with chronic obstructive pulmonary disease. *Am J Respir Crit Care Med* 2017; 196: 438–446.
